# Case of Strangulated Crural Hernia Successfully Treated by Chloroform

**Published:** 1852-05

**Authors:** Robert Burns

**Affiliations:** Frankford, Pa.


*Case of Strangulated Crural Hernia successfully treated by Chloroform.* Communicated by Robert Burns, M. D., of Frankford, Pa.

Joseph Eastburn, a middle aged German, employed in the chemical establishment of Mr. Lennig, of Bridesburg, of a spare habit but firm muscular fibre, had been afflicted with hernia for several years, and subject to diarrhœa. Not using the protection of a truss, his hernia became strangulated on Friday, the 26th inst., and being unable to reduce it as he had himself been in the habit of doing on former occasions, his family physician, Dr. Wiley, was called in, who by venesection, ice, taxis, &c., made every effort to restore the intestine, but without effect. On the evening of the 27th, Dr. Burns of Frankford was called in, when, on consultation, it was determined to bring the system under the influence of enemata of tobacco, and the internal use of tartarized antimony, and thus endeavor to reduce the hernia, which was ascertained to be a crural one. Our best directed efforts were still unavailing, and the operation being the last resort, it was arranged to send for Dr. Keller, of Philadelphia; and at early hour on the 29th, endeavor to rescue the sufferer from impending danger. By some delay in receiving the communication, Dr. K. did not arrive until after 8 o'clock A. M. In the mean time every preparation was made to operate should Dr. K. not come, or no other procedure avail. Dr. K. on examination of the patient, confirmed the diagnosis, and after considerable effort to reduce, the course was abandoned as altogether impracticable. From the tension and tenderness of the abdomen, we feared extensive intestinal and peritoneal inflammation; and before resorting to the knife, it was suggested to make a trial of chloroform; which being agreed upon, Dr. Burns hurried to Frankford, and procured a reliable article, which was used in the following manner:—A piece of muslin sufficiently large to cover the hernial tumor was saturated with the chloroform, and applied to the part, over which a small compress was placed, to retard evaporation. Of this part Dr. Keller took charge, while at the same time, Dr. Burns applied the same agent to the mouth and nares, dropped on a piece of muslin, causing the patient to inhale it as freely as possible; thrice he inhaled fresh portions, and was brought under its influence, which made him very restless with constant endeavors to push us away, and struggling to rise up. At this moment, Dr. K. made the effort at reduction, which was entirely successful, and the opening under Poupart's ligament was plainly distinguishable, so as to admit the point of the finger. Our gratification was very great, our patient spared a painful and dangerous operation, his family and friends were full of hope, and another instance was added to the multitude of the past, in favor of the wonderful effects and immense value of chloroform, in the mitigation of human suffering. Could our sketch close here, our exultation would be complete; but far otherwise was the intelligence obtained on the morning of 29th, when to our sorrow, the radiance of hope was obliterated, by the sable curtain of death. Dr. Wiley informed the writer, that the compress and bandage which were applied by Dr. Keller until a truss could be procured, were detached by the patient getting out of bed, and the hernia reappeared, but was easily reduced by him; the patient however sank rapidly, and died at 1 o'clock, A. M., on the 29th. Dr. Wiley and the writer made a post mortem examination of the abdominal cavity, and a minute dissection of the inguinal region. The peritoneum was universally inflamed, together with the greater portion of the intestines, some parts of which were gangrenous, especially that portion of the ilium which was constricted. Both peritoneal and intestinal adhesions were extensive, besides perforations of the cœcum, with of course effusions and deposits of coagulable lymph; the hernial sac, as it protruded through the crural ring, was about the size of a hickory nut, and in thickness the sixteenth of an inch, its inner surface slightly inflamed, and the point of constriction evidently at the neck under Poupart's ligament, the falciform process of fascia lata being free.

Thus, from the condition of the abdominal viscera, no means could have availed to save the patient, within the last twenty-four or thirty-six hours of his existence, at least if he had been operated on, death would have ensued, and the operation perhaps unjustly blamed. But may we not feel encouraged to expect from the use of the chloroform, at an early period of strangulated hernia, strong probability of success; as in this case it accomplished more than all other means.

